# Cemiplimab-Associated Sinusoidal Obstruction Syndrome

**DOI:** 10.14309/crj.0000000000001038

**Published:** 2023-04-20

**Authors:** Nadeen Y. Sarsour, Marta Minervini, Shahid M. Malik

**Affiliations:** 1 University of Pittsburgh Department of Internal Medicine, Pittsburgh, PA; 2 University of Pittsburgh Department of Pathology, Pittsburgh, PA; 3 University of Pittsburgh Division of Gastroenterology, Hepatology, and Nutrition, Pittsburgh, PA

**Keywords:** check-point inhibitor, veno-occlusive disease, sinusoidal obstruction syndrome, cemiplimab, nodular regenerative hyperplasia

## Abstract

A 58-year-old woman developed new-onset recurrent ascites after the recent initiation of cemiplimab for the treatment of advanced basal cell carcinoma. A comprehensive serological workup for viral, metabolic, and autoimmune causes was unrevealing. Transjugular liver biopsy demonstrated parenchymal changes consistent with a diagnosis of sinusoidal obstruction syndrome. While this is a condition commonly observed in patients after hematopoietic stem cell transplantation or use of chemotherapeutic agents, it should also be considered in patients who develop new-onset liver dysfunction after the initiation of checkpoint inhibitors.

## INTRODUCTION

Hepatic veno-occlusive disease (VOD), also known as sinusoidal obstruction syndrome (SOS), is a clinical syndrome characterized by hepatomegaly, right upper quadrant pain, and ascites that can occur after high-dose chemotherapy, hematopoietic stem cell transplantation (HSCT), or liver transplantation.^1^ The pathogenesis of this disease begins with sinusoidal endothelial injury, which leads to sinusoidal microcirculatory disturbances, sinusoidal congestion, and alterations in normal blood flow resulting in activation of hepatic stellate cells, which deposit collagen in the space of Disse causing atrophy of surrounding hepatocytes.^1^ This can eventually lead to cholestasis and elevated total bilirubin levels. Chemotherapy drugs, including granulocyte colony-stimulating factor, calcineurin inhibitors, cyclosporin, and gemtuzumab, have been linked to VOD/SOS.^2–5^ Endothelial injury mediated by cytotoxic T lymphocytes in patients with graft-vs-host disease (GVHD) can also lead to this finding.^1^ The diagnosis of VOD can be made using the European Bone Marrow Transplantation criteria in adults, which requires the following characteristics to make the diagnosis of late-onset VOD/SOS: classical SOS beyond day 21 (this includes bilirubin ≥2 mg/mL and 2 of the following: painful hepatomegaly, weight gain, and ascites), histologically proven SOS, or ≥2 of the classical criteria and ultrasound or hemodynamic evidence of SOS.^6^ Defibrotide, a drug whose mechanism is not fully understood, is the only studied drug used for the treatment of moderate and severe VOD/SOS.^7^

Cemiplimab is an immune checkpoint inhibitor, specifically a programmed cell death protein 1 (PD-1) inhibitor, recently approved for the treatment of cutaneous squamous cell carcinoma.^8^ PD-1, a protein on the surface of activated T cells is bound by PD-L1, which leads to T-cell inactivation. Cancer cells express PD-L1 leading to inhibition of T-cell activation and prevention of T-cell-mediated destruction of tumor cells. PD-1 inhibitors promote T-cell activation to generate a response against malignant cells.^8^ Immune checkpoint inhibitors have exhibited a variety of neurologic, cardiopulmonary, and gastrointestinal side effects, such as hepatitis, myocarditis, and meningitis. Regarding gastrointestinal immune-related adverse events, the most commonly known side effects include severe colitis, elevated liver function tests, hepatotoxicity, and hepatitis.^9–11^ Hepatotoxicity has been demonstrated to be a rare but known side effect of cemiplimab, specifically.^12^ This drug has been avoided in patients with solid-organ transplants because of risk of graft rejection, although the use of prophylactic steroids to prevent this complication has been explored.^13^ There is 1 documented case of checkpoint inhibitor-associated VOD/SOS associated with the medication nivolumab.^14^ There are currently no known reports of cemiplimab VOD/SOS. However, there are reports regarding checkpoint inhibitor-mediated biliary injury and nodular regenerative hyperplasia.^15,16^

This case documents a patient with diagnosis of VOD/SOS after the initiation of cemiplimab.

## CASE REPORT

A 58-year-old woman with a history of locally advanced basal cell carcinoma of the left eye treated with 6 months of cemiplimab and no preexisting liver conditions presented with new-onset ascites 1 month after cessation of this PD-1 checkpoint inhibitor. On admission, diagnostic paracentesis demonstrated a serum albumin ascites gradient greater than 1.5. Laboratory results showed the following: sodium 129 mmol/L, creatinine 0.8 mg/dL, albumin 2.9 g/dL, total bilirubin 1.2 mg/dL (direct 0.5 mg/dL), alanine transaminase 59 IU/L, aspartate aminotransferase 51 IU/L, alkaline phosphatase 884 IU/L, gamma glutamyl transferase 373 IU/L, and international normalized ratio 2.1. A viral hepatitis panel, including hepatitis A antibody immunoglobulin (Ig) M, hepatitis C antibody with reflex to RNA, hepatitis B core antibody, and hepatitis B surface antigen, was negative. Abdominal and pelvic computed tomography demonstrated diffuse heterogeneous enhancement of liver parenchyma suggesting passive congestion. Quantitative cytomegalovirus (CMV) polymerase chain reaction (PCR) was 427 IU/mL.

At this time, our patient was evaluated by oncology and started on prednisone 100 mg daily in an attempt to treat a suspected checkpoint-mediated hepatitis with no effects on alkaline phosphatase or clinical condition with worsening liver function tests. Owing to concern for CMV reactivation, a multidisciplinary plan was made with infectious disease, hepatology, and oncology to decrease her prednisone to 40 mg daily and follow the CMV viral load over time.

**Figure 1.**
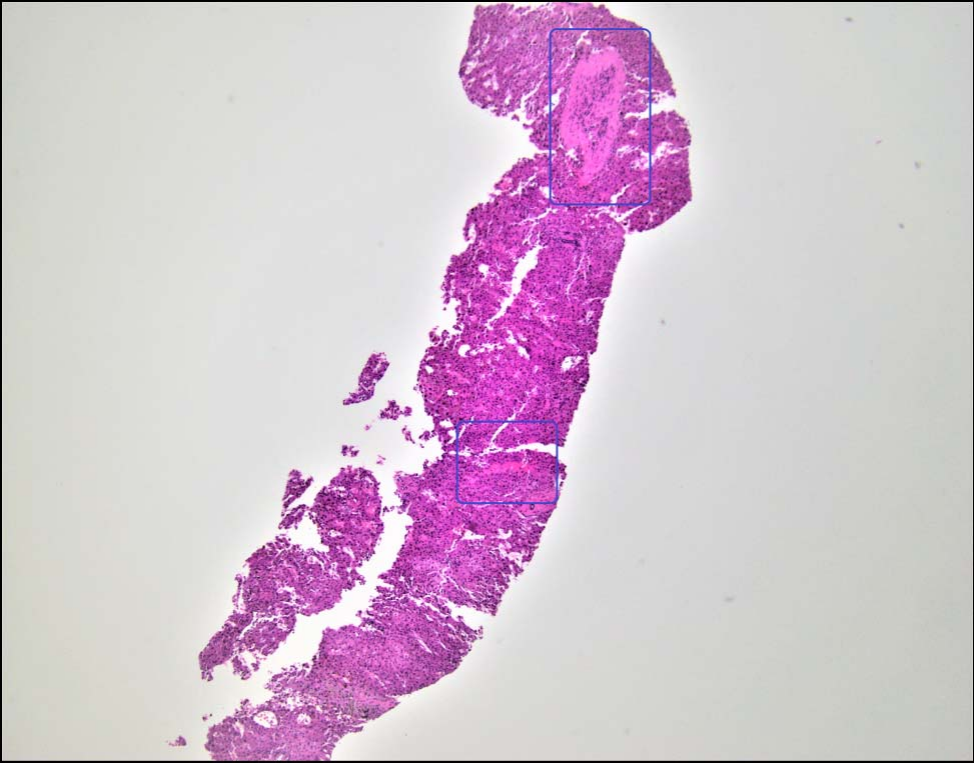
Moderate nodular regenerative hyperplasia of previously normal hepatic parenchyma.

**Figure 2.**
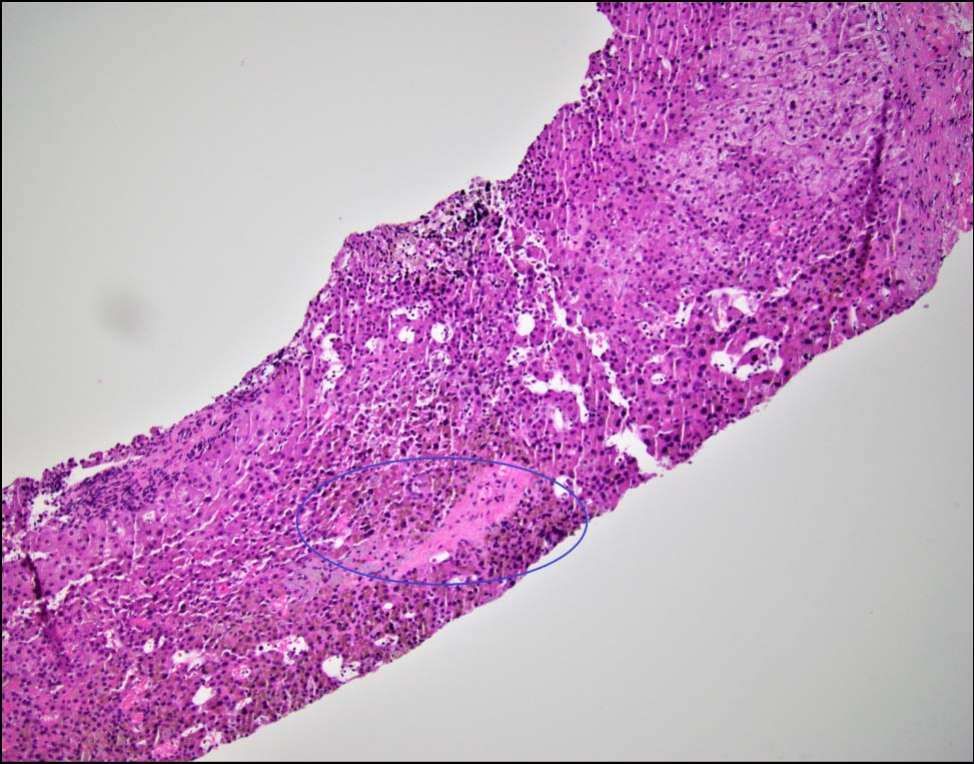
Prominent central vein sclerosis with fibrous obliteration.

**Figure 3.**
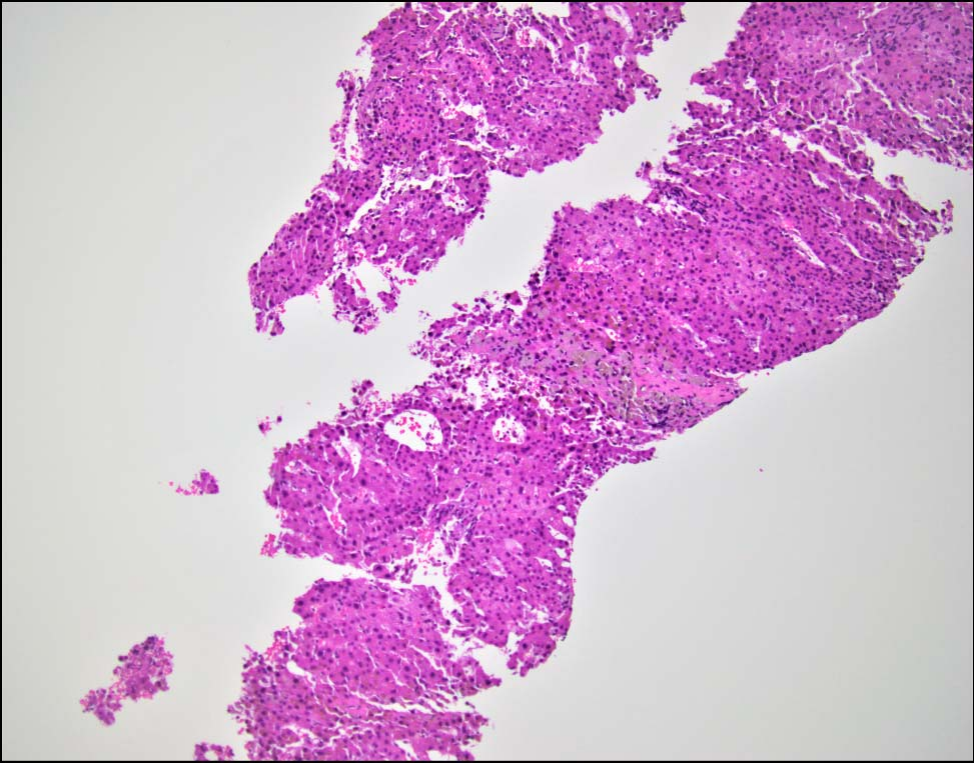
Centrizonal sclerosis with ceroid-laden macrophages. Ceroid-laden macrophages are a well-recognized pathologic feature of liver injury commonly seen in autoimmune drug-induced liver injury. Ceroid contains modified lipoproteins (such as malondialdehyde-modified low-density lipoprotein), which are generated as a consequence of chronic oxidative stress.

**Figure 4.**
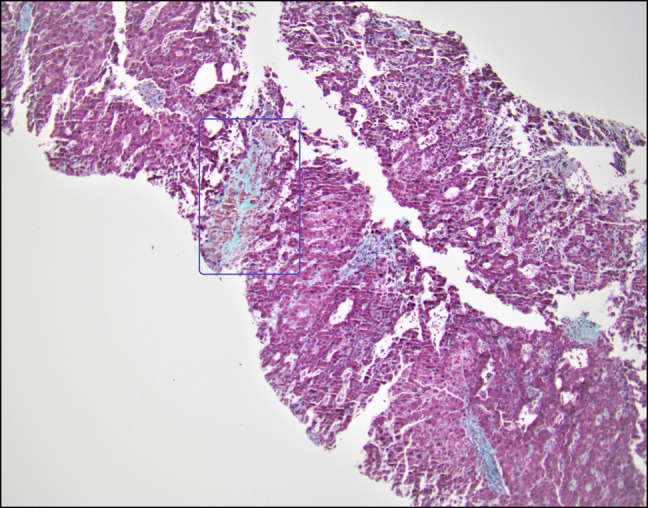
Elastic trichrome with centrizonal sclerosis.

The patient returned to our institution 1 month later as a transfer for recurrent ascites and worsening total bilirubin. On this admission, laboratory results showed white blood cell 11.1 × 10^9^/L, hemoglobin 14.3 g/dL, platelets 295 × 10^9^/L, sodium 129 mmol/L, creatinine 0.7 mg/dL, albumin 3.0 g/dL, total bilirubin 12.6 mg/dL (direct 7.3 mg/dL), alanine transaminase 220 IU/L, aspartate aminotransferase 147 IU/L, alkaline phosphatase 2,025 IU/L, and international normalized ratio 1.6. Hepatitis panel, anti-nuclear antibody, anti-mitochondrial antibody, anti-smooth muscle antibody were all negative. Repeat paracentesis demonstrated a negative culture. Quantitative CMV PCR was 330,573 IU/mL. Blood cultures had no growth; COVID and influenza tests were negative. She had an erythrocyte sedimentation rate of 8 mm/h, C-reactive protein 1.3 mg/L, low complements (C3 79 mg/dL and C4 19 mg/dL), and a normal urinalysis. Cryoglobulin, anti-proteinase 3, and myeloperoxidase antibody were negative. Beta-2 glycoprotein, anticardiolipin IgG, herpes simplex virus PCR, and Epstein-Barr virus IgG and IgM antibodies were negative. Computed tomography angiography of the abdomen and lower extremities showed no evidence of inflammation in large vessels. Doppler ultrasound demonstrated slow hepatopedal flow with patent hepatic vessels. Transjugular liver biopsy demonstrated a hepatic venous-free pressure of 7 mm Hg and a wedge pressure of 14 mm Hg consistent with mild portal hypertension. Pathology revealed moderate nodular regenerative hyperplasia changes (Figure 1), prominent central vein sclerosis with fibrous obliteration (Figure 2), signs of SOS/VOD and central venulitis with fibrotic changes, and sinusoidal portal hypertension. Figure 3 demonstrates centrizonal sclerosis with ceroid-laden macrophages, a well-recognized pathologic feature of liver injury commonly seen in autoimmune drug-induced liver injury. Figure 4 highlights pathologically increased fibrosis with elastic trichrome staining. A CMV immunohistochemical stain was negative. The patient was started on ursodiol 300 mg twice a day with no improvement in liver function tests or symptoms.

## DISCUSSION

Hepatic veno-occlusive disease, also known as sinusoidal obstruction syndrome, is a diagnosis often associated with chemotherapy drugs and T-cell-mediated destruction associated with GVHD; however, in our patient, we present the first report of checkpoint inhibitor-induced VOD/SOS.^1^ Diagnostic criteria, which include histologic findings through transjugular biopsy, elevated total bilirubin, and ascites refractory to diuretics, were all met by this patient. In addition, our patient displayed a cholestatic pattern of liver injury associated with bile duct obstruction because of the severity of endothelial damage. Diagnostic workup included a full hepatitis panel, anti-nuclear antibody/anti-mitochondrial antibody/anti-smooth muscle antibody, herpes simplex virus, Epstein-Barr virus, and CMV immunohistochemical staining on biopsy that were all negative along with no response to a prolonged steroid course ruling out all other etiologies of VOD/SOS. Of note, this patient was treated with valganciclovir for CMV reactivation with no improvement in liver function. Oncology and infectious disease consultation was requested, and it was concluded that symptoms of liver dysfunction occurred before CMV reactivation and this reactivation was induced by initiation of steroids. There was no evidence of end-organ dysfunction secondary to CMV, and the patient was ultimately diagnosed with CMV viremia separately from SOS as a consequence of cemiplimab therapy. Onset of symptoms and VOD were preceded by a 6-month course of anti-PD1 therapy for the treatment of basal cell carcinoma. While there are few reports of checkpoint inhibitor-associated VOD, the mechanism of T-cell activation may be similar to that of cytotoxic T-cell activation in GVHD.

Given the morbidity and mortality of VOD/SOS, immediate cessation of the offending agent occurred; however, the patient was transitioned to hospice and died before further treatment, such as defibrotide, could be pursued. Although a variety of immune-related adverse effects have been reported after PD-1 inhibitors, VOD/SOS has not been reported. We report on a documented case of hepatic veno-occlusive disease associated with immune checkpoint inhibition, which should be considered in a differential diagnosis of liver dysfunction in patients receiving these medications.

## DISCLOSURES

Author contributions: NY Sarsour identified the case, wrote and edited the report, and obtained the images and is the article guarantor. M. Minervini interpreted the slides, confirmed diagnosis, and selected images for the case report. SM Malik confirmed case details and wrote and edited the report.

Financial disclosure: None to report.

Previous presentation: This case was accepted for presentation at ACG Annual Meeting; October 21-26, 2022; Charlotte Convention Center, Charlotte, North Carolina.

Informed consent was obtained for this case report.
